# Harvesting multipotent progenitor cells from a small sample of tonsillar biopsy for clinical applications

**DOI:** 10.1186/s13287-017-0619-x

**Published:** 2017-07-27

**Authors:** Raju Khatri, Michal Arad, Timothy Ortlip, Benjamin A. Portney, W. Alex Meltzer, Silviu Diaconu, Lorna E. Silipino, Ying Wang, David M. Kaetzel, Rodney J. Taylor, Michal Zalzman

**Affiliations:** 1 Department of Biochemistry and Molecular Biology, University of Maryland School of Medicine, Baltimore, MD 21201 USA; 2 The Center for Stem Cell Biology and Regenerative Medicine, University of Maryland School of Medicine, Baltimore, MD 21201 USA; 3 Marlene and Stewart Greenbaum Cancer Center, University of Maryland School of Medicine, Baltimore, MD 21201 USA; 4 Department of Otorhinolaryngology-Head and Neck Surgery, University of Maryland School of Medicine, 108 N. Greene Street, Baltimore, MD 21201 USA; 5 Division of Plastic, Reconstructive, & Maxillofacial Surgery, R Adams Cowley Shock Trauma Center, Baltimore, MD 21201 USA

**Keywords:** Mesenchymal progenitor cells, Multipotency, Cell therapy, Regenerative medicine, Tonsil

## Abstract

**Background:**

Human adult stem cells hold the potential for the cure of numerous conditions and degenerative diseases. They possess major advantages over pluripotent stem cells as they can be derived from donors at any age, and therefore pose no ethical concerns or risk of teratoma tumor formation in vivo. Furthermore, they have a natural ability to differentiate and secrete factors that promote tissue healing without genetic manipulation. However, at present, clinical applications of adult stem cells are limited by a shortage of a reliable, standardized, and easily accessible tissue source which does not rely on specimens discarded from unrelated surgical procedures.

**Method:**

Human tonsil-derived mesenchymal progenitor cells (MPCs) were isolated from a small sample of tonsillar tissue (average 0.88 cm^3^). Our novel procedure poses a minimal mechanical and enzymatic insult to the tissue, and therefore leads to high cell viability and yield. We characterized these MPCs and demonstrated robust multipotency in vitro. We further show that these cells can be propagated and maintained in xeno-free conditions.

**Results:**

We have generated tonsillar biopsy-derived MPC (T-MPC) lines from multiple donors across a spectrum of age, sex, and race, and successfully expanded them in culture. We characterized them by cell surface markers, as well as in vitro expansion and differentiation potential. Our procedure provides a robust yield of tonsillar biopsy-derived T-MPCs.

**Conclusions:**

Millions of MPCs can be harvested from a sample smaller than 1 g, which can be collected from a fully awake donor in an outpatient setting without the need for general anesthesia or hospitalization. Our study identifies tonsillar biopsy as an abundant source of adult MPCs for regenerative medicine.

**Electronic supplementary material:**

The online version of this article (doi:10.1186/s13287-017-0619-x) contains supplementary material, which is available to authorized users.

## Background

Multipotent mesenchymal progenitor cells (MPCs) can give rise to several types of specialized cells [1]. These fibroblast-like cells were first identified and isolated from the bone marrow (BM) and spleen, and were termed “bone marrow stromal stem cells” [2]. Presently, many other tissues such as the placenta, amniotic fluid, umbilical cord, adipose tissue, tonsils, and endometrium have been identified as sources of MPCs [3–10]. Nevertheless, the phenotype and differentiation potency of MPCs vary with respect to the tissue source from which they are isolated and the harvesting procedure [11–13]. MPCs possess the potential to differentiate into multiple cell types including adipocytes (fat), chondrocytes (cartilage), and osteoblasts (bone) [14]. Characterization of MPCs includes adherence to standard tissue culture plastic, expression of various surface antigens, and in vitro differentiation potential [15]. Like other types of adult progenitor cells, MPCs remain quiescent (nondividing) in vivo for long periods. When activated, MPCs divide and differentiate to replace injured cells as well as secreting factors to prevent inflammation and promote tissue repair [16].

Cellular therapies hold great potential for the cure of a wide range of diseases and provide enhanced treatment modalities including immunomodulatory therapies, tissue regeneration, and cancer therapies. Using MPCs is an attractive approach for cell therapy as it avoids the ethical and practical issues of embryonic- and fetal-derived stem cells [17]. Currently, many clinical trials are testing MPCs obtained from different tissue sources for the cure of numerous conditions such as autoimmunity, heart disease, bone and cartilage disease, cancer, neuropathologies, and gastrointestinal diseases [18]. Furthermore, other than disease treatment, human MPCs are equally used in drug discovery applications as replacements for primary cells and animal models for initial toxicity and screening of new compounds [19].

MPCs, also classified as pericytes, reside on blood vessels [20–22] and, therefore, the more vascularized the tissue is, the more rich the tissue is with MPCs. However, pericytes do not share a common embryonic precursor (reviewed in [23]). Moreover, recent studies have shown that pericytes from different anatomical sites, regarded as “MPCs”, differ widely in transcriptomic signatures and differentiation potential [24]. Therefore, the tissue source and the derivation procedure can affect the abundance, phenotype, and differentiation potency of MPCs [3, 11–13, 25]. Historically, BM has been one of the major sources of MPCs. However, the derivation of progenitor/stem cells from patients and healthy donors is not always possible. BM extraction requires hospitalization and leads to considerable donor morbidity, including pain and bleeding, and other complications such as infection and risk for viral exposure [26, 27]. Therefore, efforts have been made to find alternative sources of MPCs for cell therapies. Another current major source for MPC derivation is adipose tissue. However, 100 ml of tissue and blood collected from lipoaspirates provides approximately 10^5^ cells [28]. This is likely due to the mechanical trauma to the tissue incurred during the liposuction procedure, leading to a low yield and reduced cell viability [29]. Importantly, in order to achieve enough viable cells in the scale needed for clinical purposes, a large quantity of starting tissue material is needed which may result in considerable risk and donor morbidity. Therefore, for translational purposes, an accessible alternative source of MPCs is still needed.

Tonsils retrieved from tonsillectomy procedures have been shown to be a good source of MPCs [30]. For translational purposes, cells that can be retrieved from healthy donors without the risk of major complications and donor morbidity are preferred as a reliable source for cell therapies. To this end, we developed a procedure to generate highly proliferative multipotent progenitor/stem cells from a small fragment of normal tonsillar tissue. Tonsils are lymphoid tissue anatomically located at the entrance of the pharynx. Here we report a novel procedure for the isolation of MPCs from a biopsy-sized sample of human tonsil. We have isolated tonsillar MPCs (T-MPCs) from multiple donors across a spectrum of age, sex, and race, and successfully expanded them in culture. We characterized them by cell surface markers, as well as by in vitro expansion and differentiation potential. Overall, our study highlights tonsillar biopsy as an excellent source of MPCs and a viable alternative to currently used sources such as bone marrow and adipose tissue.

## Methods

### Isolation and expansion of MPCs from human tonsils

**Fig. 1:**
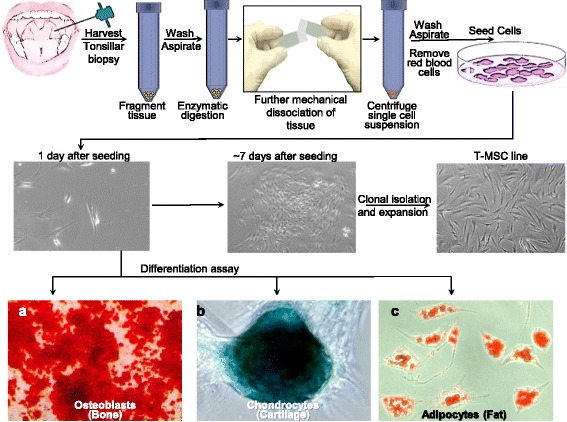
A schematic workflow of T-MPC isolation from human tonsillar biopsy. Our results show that tonsillar-derived MPCs can be expanded in culture and differentiated into various lineages including: **a** osteoblasts (bone cells), **b** chondrocytes (cartilage), and **c** adipocytes (fat cells). *T-MSC* tonsillar mesenchymal stem cell

The studies described herein were approved by the University of Maryland, Baltimore, institutional review board (IRB), with informed consent obtained from patients (IRB protocol number HCR-HP-00062781-1). A tonsillar biopsy was taken at the time of tonsillectomy and the tissue was then processed as demonstrated in Fig. 1. Fresh tonsil tissue specimens were obtained from the University of Maryland Medical Center, with an average weight of 0.88 ± 0.1 (average ± SEM) g of tissue per specimen. Tonsil specimens were kept in sterile conditions and the procedure was performed under a biological hood. The tissue was washed twice with Dulbecco’s phosphate-buffered saline (DPBS; Life Technologies) followed by washing with Iscove’s modified Dulbecco’s medium (IMDM; Hyclone). The tissue was then fragmented in a 6-cm sterile culture plate into very small pieces in IMDM medium. Minced tissue was collected into a 50-ml tube, centrifuged at 500 rpm for 1 min and washed repeatedly with DPBS until no more blood was visible. The tissue was then incubated for 45 min at 37 °C in 10 ml IMDM containing 1.6 U/ml Liberase (Roche) and 100 μg/ml DNAase (Sigma), with additional time added as needed to achieve complete digestion. At all stages of this protocol, the tube cap was not tightened to allow air into the tube and to prevent hypoxic conditions. To maximize yield, tubes were agitated every 15 min. Remaining tissue fragments were subjected to further disruption by application of mechanical force between the rough label sides of two frosted microscope slides, followed by passage through a pipette to allow release of single cells. This step was repeated until no tissue fragments were visible. Cells were collected into 15-ml tubes and washed with 5 ml DPBS, followed by centrifugation at 500 rpm for 5 min. The cell pellet was transferred to a fresh tube until a clean pellet was achieved. Cells were suspended in fresh T-MPC medium (500 ml Dulbecco’s modified Eagle’s medium (DMEM; Life Technologies), 10% heat inactivated fetal bovine serum (FBS; Sigma), 1× GlutaMAX (Invitrogen), 1% sodium pyruvate (Invitrogen), 1% nonessential amino acids (Invitrogen), 1% penicillin/streptomycin (Life Technologies), and 100 μM beta-mercaptoethanol (Life Technologies)). Cells were then passed through a 70-μm nylon mesh filter. Cells were stained with trypan blue to determine cell viability. Five million live cells were seeded per 10-cm plate (total 10 plates) per donor. The remaining cells were aliquoted to 5 million cells per vial and were taken for cryogenic preservation. The next day, cells were washed thoroughly to remove nonadherent cells and the medium was replaced with fresh T-MPC medium. Visible individual colonies were typically formed within 1 week. Clones were dissociated by Accutase, isolated, and seeded separately in one well of a six-well plate to make passage 1 (P1). To keep the culture potential and to avoid loss of clones due to aging, clones were randomly taken per patient for further analysis, and the remainder of the clones frozen at P1. Clones were then continuously maintained by subculturing at low densities of 10^5^ cells per 10-cm plate and harvested at 70% confluency thereafter.

### Population doubling assay

The cells attached after tissue harvesting were considered passage zero (P0), with passage number corresponding to the number of times the cells were subcultured. For each culture passage, 2.5 × 10^4^ T-MPCs per well were seeded in six-well plates in triplicate. Cells were harvested by accutase (Millipore) every 5 days to ensure cells were constantly grown in subconfluence conditions. Upon harvesting by accutase, cells were counted and 2.5 × 10^4^ cells were reseeded in six-well plates in triplicate. Cells were continuously subcultured until the cells stopped replicating and culture reached cellular senescence. The cumulative population doublings (PD) are the total number of times the cell population have doubled during subculture and are calculated by continuously adding the PD per each passage. The number of PD per donor was calculated using the formula: $PD=\mathrm{L}\mathrm{n}\;\left(\frac{\mathrm{N}\mathrm{t}}{\mathrm{N}0}\right)\times 3.33,$ where N0 is the number of cells at seeding and Nt is the number of cells counted at harvesting.

### Population doubling assay and doubling time in xeno-free medium

To culture T-MPCs in xeno-free and serum-free conditions, culture plates were first precoated with 20 μg/ml fibronectin in phosphate-buffered saline (PBS). T-MPCs were seeded and passaged once every 7 days in fibronectin-coated (Thermo) 12-well plates at approximately 10% confluence (3500 cells per well). Cell growth rate was calculated as above. The doubling time (Td) was calculated as the log2 of the duration of culture (h), divided by the log(final cell number) minus log(number of cell seeded): Td = $\frac{ \log (2)}{ \log \left(1+r\right)}$ X $\frac{24h}{time\;(h)}$.

### Flow cytometry analysis

T-MPCs were harvested using accutase from 70% confluent plates. Cells were fixed in 4% paraformaldehyde for 10 min and samples were taken for immunofluorescence staining and incubated with corresponding antibodies for the MPC markers CD44-FITC, CD90-PE, CD73-PE, and CD105-Alexa 488. The following negative markers were excluded: CD45-Alexa 647, CD31-Alexa 594, HLA-DR-Alexa 647, and CD19-Alexa 647 (Bioloegend; 1:100 dilution) in blocking solution (10% FBS, 1% bovine serum albumin (BSA) in DPBS) on ice for 30 min. Controls used were cells incubated with rabbit IgG, mouse IgG, and rat IgG with corresponding chromophores. Additional negative controls used were cells incubated in blocking solution without primary antibody. After three washes with blocking solution, cells were washed with DPBS and analyzed by flow cytometry.

### Flow cytometry for pluripotent stem cells markers

T-MPCs were harvested and fixed as described above and incubated with corresponding antibodies: SSEA4 (1:100), TRA-1-81 (1:250), and TRA-1-60 (1:250) (Cell Signaling) in blocking solution (10% FBS, 1% BSA in DPBS) on ice for 30 min. Mouse embryonic stem cells were used as positive controls. Negative control MPCs were incubated with mouse IgG or rat IgG with the corresponding secondary antibodies. Following washes with blocking solution, cells were mixed with the corresponding secondary antibody in blocking solution and incubated for 20 min on ice. Cells were washed with DPBS and analyzed by flow cytometer.

### Osteogenic differentiation assay

To induce differentiation toward the bone lineage, osteogenic media was prepared using phenol red free DMEM (Life Technologies), supplemented with 50 μm/ml l-ascorbic acid 2-phosphate (Sigma), 10 mM β-glycerophosphate (Sigma), 10 nM dexamethasone (Sigma), 1% penicillin and streptomycin (Life Technologies), and 10% FBS. Twenty thousand (2 × 10^4^) T-MPC cells were seeded per well in 24-well plates. Next day, cells were washed with DPBS, followed by the addition of complete medium (CM) or osteogenic medium to the corresponding wells. The medium was changed every 4 days and cells were differentiated for 21 days.

### Alizarin Red S Staining

To validate osteogenic differentiation, calcium deposits can be demonstrated by Alizarin Red S staining. Twenty thousand cells (2 × 10^4^) were seeded on 24-well plates. A day after seeding, cells were washed with DPBS and then allowed to grow in either CM or osteoblast differentiation medium (ODM) for 28 days. Calcium deposits are an indication of successful differentiation of MPC into osteoblasts. Cells differentiated under osteogenic conditions and undifferentiated controls cells were fixed by incubating with 10% formaldehyde at room temperature for 30 min. Cells were stained with 2% Alizarin Red S solution (AMRESCO) in distilled water (pH 4.4–4.3) at room temperature in the dark for 45 min and then washed with distilled water. Samples were photographed to visualize bright orange-red color in calcified osteoblasts.

### Chondrogenic differentiation assay

To induce cartilage differentiation of T-MPCs, cells were harvested and centrifuged at 500 rpm for 5 min to generate cell pellets of 2 × 10^5^ cells in chondrogenesis differentiation medium (StemPro, chondrogenesis differentiation kit, Life technologies). Cell colonies presented proteoglycans within about 7–21 days after seeding.

### Alcian blue 8GX staining

Differentiated cells treated with chondrogenic medium and undifferentiated control cells were fixed by incubating with 4% paraformaldehyde at room temperature for 10 min. The cells were washed twice with PBS, gently rinsed with ddH_2_O, and stained with Alcian blue 8GX solution (Fluka analytical) for 30 min at room temperature. Then stained cells were washed with running tap water for 2 min, rinsed with distilled water, and staining was documented using a phase contrast microscope to demonstrate proteoglycans in blue.

### Adipogenic differentiation assay

To differentiate T-MPCs into adipocytes, adipogenic medium was made using phenol red free DMEM, supplemented with 10% FBS, 3-isobutyl-1-methylxanthine (0.5 mM), dexamethasone (1 μM), insulin (1 μg/ml; Sigma), and 1% penicillin/streptomycin (Life Technologies). Twenty thousand (2 × 10^4^) T-MPCs were seeded on 24-well plates. The next day, attached T-MPCs were washed with PBS, and 1 ml of freshly prepared adipogenic medium was added to each well. Medium was changed every 4 days. Cells were allowed to grow in adipogenic medium for 3 weeks.

### Oil red O staining

Cells differentiated in adipogenic medium and undifferentiated control cells were fixed with 4% paraformaldehyde for 10 min at room temperature, washed twice with PBS, and incubated with 2 ml 60% isopropanol at room temperature for 5 min. Isopropanol solution was discarded and a 2-ml working solution of Oil Red O (Sigma) was added to each well and cells were incubated for 5 min at room temperature. The cells were rinsed with tap water until all residual stain was removed and the water was clear. Lipids droplets appear red and stained cells were demonstrated using a phase contrast microscope.

### Total RNA extraction and generation of cDNA

Total RNA was extracted from 10^6^ differentiated or undifferentiated control cells using the Qiagen RNeasy mini Kit, following the manufacturer’s protocol. For each sample, reverse-transcription polymerase chain reaction (PCR) was performed using 1 μg of total RNA to reverse transcribe in cDNA by Superscript III (Invitrogen) following the manufacturer’s instructions.

### Real-time quantitative PCR analyses for differentiation markers

To define upregulation in differentiation related genes by real-time quantitative PCR (qPCR), 10 ng cDNA of each sample was used per reaction in triplicate, using SYBR green I Master solution (Roche) and following the manufacturer’s protocol. A LightCycler 480 II machine (Roche) was used for qPCR analysis. Fold induction was calculated by the delta-delta Ct method using 200 ng of each primer to determine the target gene expression. Primers used in this study are listed in Table 1.

**Table 1:** A list of osteoblast, adipocyte, and chondrocyte primers

	Primer	Forward (5’–3’)	Reverse (5’–3’)
Osteoblast markers	BMP2	CCCTACATGCTAGACCTGTATC	GTTGTTTTCCCACTCGTTTCTG
	OPN	CCTTCCAAGTAAGTCCAACGAA	GACAACTGGAGTGAAAACTTCG
	OCN	ATGAGAGCCCTCACACTCCTC	GCCGTAGAAGCGCCGATAGGC
	ALP	TGGAGCTTCAGAAGCTCAACACCA	ATCTCGTTGTCTGAGTACCAGTCC
	RUNX2	TTTAGGGCGCATTCCTCATC	GGAGGGCCGTGGGTTCT
Osteocyte markers	FGF23	TTGGATCACACTATTTCGACCC	GAAGTGAATTAGGGGGATCTCG
	DMP1	TCTTTGTGAACTACGGAGGGTA	TGAGCCAAATGACCCTTCCA
	MEPE	GAGGAAAAGGTAGACTGAGATTCT	GGGACAAATCTTTCTTTCTTTCCT
	SOST	CAAGAATGATGCCACGGAAATC	GGACACGTCTTTGGTCTCAA
Control	RPLP0	CAGCAAGTGGGAAGGTGTAATCC	CCCATTCTATCATCAACGGGTACAA
Adipogenic markers	ACAN	TGATGTTCCCTGCAATTACCAC	CAAAAAGCGACAAGAAGAGGAC
	Leptin	ATTTTCAGAAGAGAACGGACATTC	TGCTCCCCTTCTTCAAAATGTA
Chondrogenic markers	ATP2A2	AACTACCTGGAACCTGCAATAC	GGGTTGGTAGATGTGTTGCTAA
	COL10A1	GAGTAAAGGTATAGCAGTAAGAGGA	CATATGGTCCTCTCT CTCCTGG
	PPRAG	AAGACAACAGACAAATCAACCG	GTCTTCTTGATCACCTGCAGTA

*ALP* alkaline phosphatase, *BMP2* bone morphogenetic protein 2, *DMP1* dentin matrix protein 1, *FGF23* fibroblast growth factor 23, *MEPE* matrix extracellular phospho-glycoprotein, *OCN* osteocalcin, *OPN* osteopontin, *RUNX2* runt-related transcription factor 2, *SOST* sclerostin

### Telomerase activity measurement

All the cell lines were cultured in triplicate in complete medium and harvested after 2 days. Cell lysates were prepared from 10^6^ cells per sample. Telomerase activity was measured by TRAP assay using a TRAPEZE Telomerase Detection Kit (Millipore) according to the manufacturer’s instructions. Telomerase positive controls used were Tu167 cancer cells and HeLa cells. Technical negative controls used were heat inactivated extracts per each sample. Results are shown as mean ± SEM in three biological replicates obtained from three independent experiments. Data were analyzed by two-way analysis of variance (ANOVA).

### Teratoma formation assay

Teratoma forming assay was preformed using subcutaneous engraftment of 2 x 10^6^ T-MPCs expressing GFP in NOD/SCID gamma immunodeficient mice (*n* = 10). Cells were harvested by accutase and prepared for injection in DPBS. Mice were monitored every 3 days for seven months for teratoma formation. Upon termination of the study, the fat pad tissues in the injection region was excised and examined for evidence of teratoma formation. Mice were thoroughly examined at the experimental endpoint and teratoma formation or migration from the primary injection site was excluded. The GFP reporter gene allowed us trace the cells upon the completion of the experiment. The human specific Anti-human HSP27 (NeoMarkers; 1:1000 dilution) was used to locate the cells and the point of injection by immunofluorescence.

### Statistical analysis

Student’s *t* tests were performed to assess a significant difference in the fold change between differentiated cells and undifferentiated T-MPCs for each of the markers. These analyses were performed using Graph pad Prism. Results were considered to be statistically significant when *p ≤* 0.05. The software STATSTICA 13 and GraphPad Prism 5 were used for data analyses and formation of the figures.

## Results

### Derivation of adult multipotent MPCs from a small fragment of tissue

The ideal source for an autologous graft, or for the generation of universal donors, is a tissue specimen that can be retrieved without the risk of major complications and donor morbidity. To this end, we developed a procedure to generate highly proliferative multipotent MPCs from a small sample of tonsillar tissue. We have successfully generated T-MPC cell lines from 14 donors (9 females and 5 males). These samples were distributed across three age groups: pediatric (age 3–12 years; *n* = 7), young adults (age 20–35 years; *n* = 5) and middle-aged (age 40+ years; *n* = 2) donors. To ensure proper tissue handling and to achieve optimal results, tonsil tissue samples were put in a sterile vessel on wet ice and transferred to the laboratory within hours from tissue harvesting. However, even when samples were stored at 4 °C for up to 24 h, no significant change in yield was observed. The weight of the sample was recorded on arrival at the laboratory. The tissue then was minced in IMDM medium, enzymatically digested, and any remaining fragments were mechanically dissociated until single cells were released to the medium. Cells were cultured to establish T-MPC lines (Fig. 1). We generated T-MPCs from samples that averaged 0.88 ± 0.1 g (mean ± SEM) with a high yield and efficiency. To test MPC isolation efficiency compared to traditional methods using collagenase type I [7, 30, 31] digestion for 30 min, fresh tonsillar tissues from three donors were used and compared for cell viability and yield. Our results indicate that digestion with collagenase I in combination with DNAase I leads to a partial digestion and poor cell viability <50% following 30-min digestion. Conversely, using Liberase and DNAase I incubation for >45 min leads to an average cell viability of >90%. The number of cells isolated per 1 g of tissue ranged from 0.2–1 billion cells with an average of 4.6 × 10^8^ ± 5.4 × 10^6^ (mean ± SEM) cells/g. Next, we seeded samples of 5 million cells per 10-cm plate followed by overnight incubation at 37 °C in a 5% humidified incubator. Approximately 2% of the total tonsillar cells adhered to the plate. Attached cells were allowed to grow to form colonies and clones were isolated to establish P0. Therefore, within a week and prior to the first cell split, 1 g of tonsillar tissue yields an average of 6.2 × 10^7^ ± 4.4 × 10^6^ (mean ± SEM) T-MPCs. These T-MPCs can then be further massively expanded in culture.

### T-MPCs express mesenchymal progenitor cell markers

**Fig. 2:**
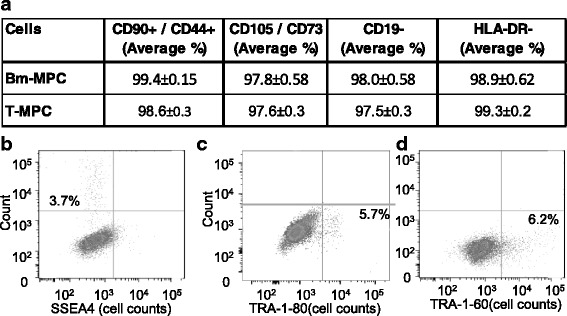
Characterization of T-MPCs by immunostaing and flow cytometry. **a** Analysis of costaining demonstrates that our T-MPCs are CD44 and CD90, CD73, and CD105 positive and CD19 and HLA-DR negative. *n* = 14 donors in triplicates. *Bm-MPC* bone marrow-derived mesenchymal progenitor cell, *T-MPC* tonsillar mesenchymal progenitor cells. **b** A fraction of our T-MPCs express the pluripotency related markers SSEA4, **c** TRA-1-80 and **d** TRA-1-60

Flow cytometry analysis of MPC markers in T-MPCs from all 14 donors show that our T-MPCs are 99.4% ± 0.15 (mean ± SEM) positive for CD44 and CD90, 97.6% ± 0.3 (average ± SEM) positive for CD105 and CD73, and negative for CD45, CD19, HLA-DR, and CD31 (*n* = 14 donors in triplicates) (Fig. 2a; Additional file 1: Table S1). Furthermore, our results show tonsillar biopsy provides a high yield and purity of multipotent MPCs in comparable purities and markers to BM-derived MPC controls (Fig. 2a; Additional file 1: Table S1). Interestingly, we also detected a subpopulation of T-MPCs expressing the pluripotency-related surface markers SSEA-4 (3.7%), TRA-1-80 (5.7%), and TRA-1-60 (6.2%). These data suggest there is a possibility to isolate and propagate an MPC population of higher potency from this tissue.

### T-MPCs are highly proliferative

**Fig. 3:**
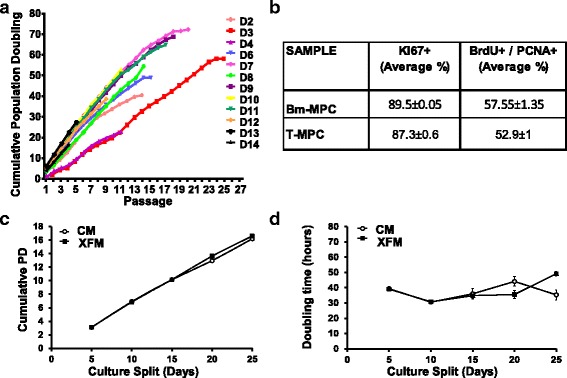
Growth curves are shown for T-MPC lines. Cells were split, counted, and 10^5^ tonsillar mesenchymal progenitor cells (*T-MPCs*) were plated per passage. **a** Growth curves demonstrate cumulative population doubling (*PD*) per individual donors. Cells were split, counted, and 10^5^ T-MPCs were plated per cell split. **b** Flow cytometry analyses with KI67 which marks dividing cells and the S-phase markers PCNA and BrdU show an actively dividing T-MPC population, which is comparable to bone marrow-derived MPCs (*Bm-MPC*). **c** Population doublings of T-MPC lines in xeno-free medium: growth curves demonstrate cumulative PD in complete T-MPC medium (*CM*) containing bovine serum compared to cells grown in xeno-free medium (*XFM*). **d** Population doubling time in xeno-free medium ranges from 31 to 35 h for at least four cell splits (20 days in culture)

To determine the in vitro expansion efficiency per donor, we performed PD assays. We seeded 2.5 × 10^4^ T-MPCs per well in triplicate in six-well plates and cells were split every 5 days to determine PD. We show a robust and highly proliferative population of cells from all donors. The majority of our T-MPC lines presented constant proliferation rates for at least 15 passages with some extending beyond 19 passages. Cumulative PD ranged from 40 to 69 PDs (Fig. 3a). To find out whether our T-MPCs present active telomerase, telomerase activity was measured by TRAP assay using a TRAPEZE Telomerase Detection Kit. Consistent with our observation of finite population doublings of at least 40 population doublings, our results indicate that our T-MPCs are telomerase negative. We next analyzed our T-MPCs by flow cytometry using the proliferation marker KI67 in cells from all 14 donors. Our results show that 87.3 ± 0.6% (mean ± SEM) of the cells actively proliferate. To study the fraction of the cells in S phase, we incubated the cells with BrdU and performed flow cytometry using anti-BrdU in combination with the S phase marker PCNA (Fig. 3b; Additional file 1: Table S1). Our data show T-MPC proliferation is comparable to BM-derived MPCs (Fig. 3b; Additional file 1: Table S1).

### Expansion of T-MPCs in xeno-free medium

We next sought to determine the expansion potential of T-MPCs in xeno-free medium. In order to avoid risks of viral cross contamination and to increase reproducibility of expansion procedures, cells were required to efficiently grow in the absence of animal-derived products and in defined medium. Therefore, we also tested our cells in xeno-free medium with defined soluble factors. Our results show that our T-MPCs can efficiently grow for at least 20 days in predefined culture conditions to achieve 18 population doublings representing a fold increase of 2.6 × 10^5^ and demonstrating a massive cell expansion capacity of T-MPCs in xeno-free medium (Fig. 3c and d).

### Osteogenic differentiation of T-MPCs

**Fig. 4:**
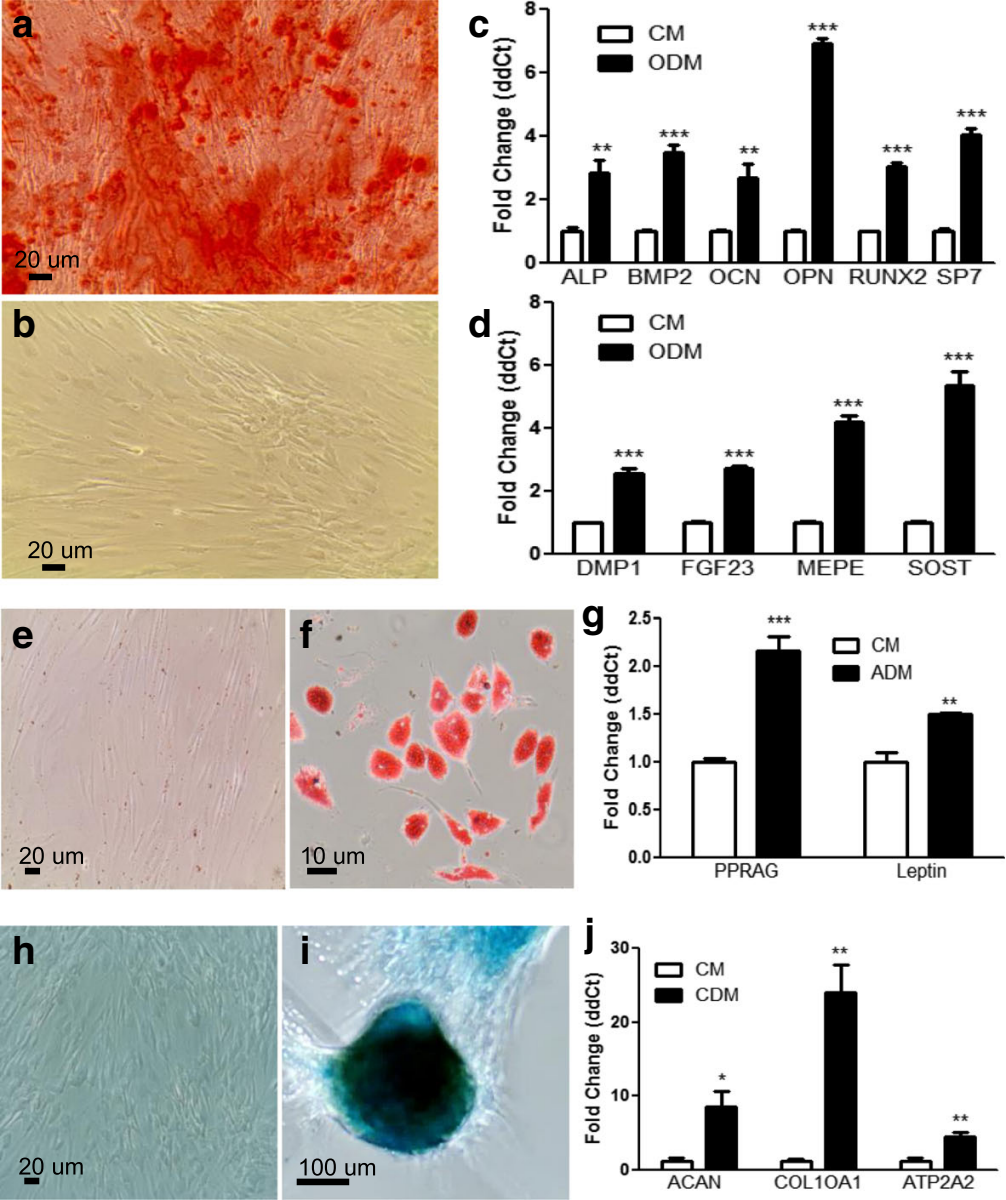
Osteogenic, chondrogenic, and adipogenic differentiation of T-MPCs. **a** Alizarin red S staining demonstrates calcium deposits in T-MPCs treated with osteogenic differentiation media (*ODM*). **b** Undifferentiated T-MPCs grown in complete media (*CM*) are negative. Real-time qPCR analysis validates the differentiation of T-MPCs in osteogenic media and shows a significant increase in expression of **c** osteoblast markers and **d** osteocyte markers compared to control. **e** Oil red O is negative in the control cells incubated at the same time and for the same duration in complete medium, while **f** Oil droplets in T-MPCs differentiated in adipogenesis medium (*ADM*). **g** Real time RT-qPCR shows T-MPC-derived adipocytes express high levels of the adipogenic-specific genes PPRAG and Leptin compared to undifferentiated controls in CM. **h** Alcian blue staining is negative in T-MPCs grown in CM, and **i** T-MPC-derived chondrocytes grown in chondrogenesis media (*CDM*) stained in blue. **j** RT-qPCR confirms an increase in the chondrogenic-specific markers ACAN, COL10A1, and ATP2A2 following differentiation compared to undifferentiated T-MPC controls. All results are presented as mean ± SEM obtained in triplicate from donors in multiple independent experiments. *p ≤ 0.05, **p ≤ 0.01, ***p ≤ 0.001. *ALP* alkaline phosphatase, *BMP2* bone morphogenetic protein 2, *DMP1* dentin matrix protein 1, *FGF23* fibroblast growth factor 23, *MEPE* matrix extracellular phospho-glycoprotein, *OCN* osteocalcin, *OPN* osteopontin, *RUNX2* runt-related transcription factor 2, *SOST* sclerostin

Differentiated osteoblasts present massive extracellular calcium deposits in vitro. To define the osteogenic differentiation potency in vitro of our T-MPCs, cells were grown in osteogenic differentiation medium for 21 days. Following osteogenic induction, the morphology of our MPCs dramatically changed from the fibroblastic phenotype to the expected more flattened type. Our results show that differentiated cells are positively stained for Alizarin Red S (Fig. 4a), suggesting osteogenic differentiation and extracellular calcium accumulation. Control T-MPCs grown at a similar density for the same time duration in CM were negative (Fig. 4b). Furthermore, our RT-qPCR results validate the osteogenic differentiation as we observed a significant upregulation in the osteoblast markers alkaline phosphatase (ALP), bone morphogenetic protein 2 (BMP2), osteocalcin (OCN), osteopontin (OPN), runt-related transcription factor 2 (RUNX2), and osterix (SP7) (Fig 4c). Remarkably, following osteogenic differentiation, our results indicate a significant upregulation of more mature osteocyte markers such as dentin matrix protein 1 (DMP1), fibroblast growth factor 23 (FGF23), matrix extracellular phospho-glycoprotein (MEPE), and sclerostin (SOST) (Fig. 4d). Our data suggest that T-MPCs may form early osteoblasts and that they progress to form mature osteocytes in culture.

### Adipogenesis of T-MPCs

To assess the adipogenesis potential, T-MPCs were grown in adipogenic medium for 21 days. Cells at the same density and for the same culture duration were incubated in CM and used as controls. Major morphological changes were apparent within 5 days of adipogenic differentiation, and typical oil vesicles were observed following 10 days to 2 weeks. Oil Red O staining (Fig. 4e and f) indicated accumulation of oil droplets consistent with adipogenic differentiation. Our RT-qPCR analyses further validate that T-MPC-derived adipocytes express increased levels of the adipocyte markers PPRAG and Leptin (Fig. 4g). These results indicate efficient differentiation of our T-MPCs to the adipogenic lineage.

### Chondrogenic differentiation of T-MPCs in vitro

Next, to determine differentiation toward cartilage tissue, T-MPCs were grown as attached cell pellets [32, 33] in chondrogenic medium for 21 days. T-MPC controls grown in complete medium for the same time were used as controls and showed no staining (Fig. 4h), while T-MPC-derived chondrocytes were heavily stained with Alcian blue (Fig. 4i). Total RNA extracted from the differentiated chondrocytes was used to determine the levels of chondrogenic markers. Our qPCR data validate the differentiation and a significant increase in the chondrogenic markers ACAN, COL10A1, and APT2A2 (Fig. 4j). These data indicate effective differentiation of our T-MPCs to the chondrogenic lineage.

### T-MPCS survive in vivo but do not form teratoma tumors

**Fig. 5:**
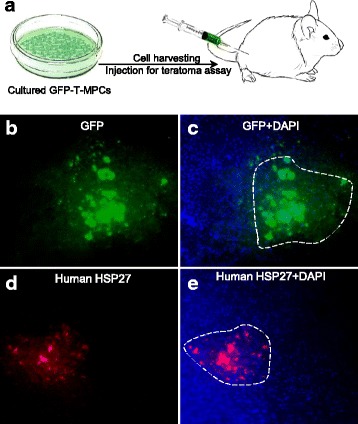
Teratoma assay of engrafted T-MPCs cells in vivo show no teratoma in mice. Schematic illustration of teratoma assay performed in NOD-SCID gamma (NSG) mice. **a** Cells are subcutaneously injected into the fat pat. Following 7 months of monitoring the mice, no teratoma tumors were formed. Fat pad was harvested for whole-mount immunostaining. Fluorescence microscope images of **b** green fluorescent protein (GFP) and **c** GFP combined with DAPI stain show MPC nuclei in the injected mouse fat pad. **d** Immunostaining assay for the human-specific antibody HSP27 (*red*) show that cells are engrafted and survived for months after, but did not form teratoma. **e** Same image as **d**, with DAPI to demonstrate nuclei. *White line* indicates the grafted cells in the injection area. *T-MPC* tonsillar mesenchymal progenitor cell

Unlike pluripotent stem cells, MPCs possess no teratoma-forming potential as the cells respond to contact inhibition in vivo and stop dividing upon transplantation. To find out if our T-MPCs replicate in vivo we generated green fluorescent protein (GFP)-positive T-MPCs. The GFP reporter gene allowed us trace the cells upon the completion of the experiment. Cells were harvested by accutase and resuspended in PBS for injection. A total of ten immunodeficient NOD-SCID gamma mice (8–12 weeks old) were subcutaneously injected with 2 × 10^6^ T-MPCs per mouse. All mice were monitored for a period of 7 months. Upon termination of the experiment, mice were thoroughly examined and no teratomas were detected in vivo. Cell engraftments in the fat pads were fixed in paraformaldehyde and taken for GFP assessment (Fig. 5a and b) and whole-mount immunofluorescence staining with the human-specific antibody anti-HSP27 (Fig. 5c and d). Our results demonstrate that our T-MPCs efficiently survived and persisted in the injection site for a prolonged period of time and did not form teratoma tumors.

## Discussion

Previous protocols to generate multipotent progenitor cells (MPCs) from tonsils are limited to discarded tissue following tonsillectomy. Furthermore, the enzymatic digestion by collagenase, accutase, or trypsin leads to incomplete tissue dissociation and a low yield [9, 30, 34]. Here, we demonstrate the extraction of MPCs from a small sample of tonsillar tissue (0.88 cm^3^) which can be obtained by a minimally invasive procedure to produce millions of MPCs. We show that our T-MPCs can be massively expanded in culture. Extraction of a small sample of less than 1 g is equivalent to a tonsillar biopsy and will avoid incisions on the body, and thus minimize the risk of infection. Additionally, harvesting MPCs from a biopsy-sized tissue fragment eases the requirement for a major surgical procedure and the resultant morbidity to the patient. The procedure can be done in the doctor’s office in minutes without the need for general anesthetics or hospitalization. It will require a topical and local anesthetic similar to that of a dental procedure and can be done in a fully awake and healthy donor.

Currently, hundreds of clinical trials are in progress exploring applications of adult MPCs for the treatment of numerous human diseases and conditions. However, extensive use of MPCs is limited at present by the low abundance and viability of the cells obtained during tissue harvesting and the invasive nature of the current procedures [28, 29, 35, 36]. Current protocols still rely on tissues discarded during surgical procedures, making mass production and even autologous cells difficult to obtain.

We have isolated, characterized, and demonstrated the robust multipotency of human tonsil-derived MPCs, with minimal mechanical and enzymatic insult to the tissue. We further show that these cells can be propagated and maintained in xeno-free conditions. Harvest and culture in xeno-free medium will render these cells suitable for cellular therapies and reconstructive procedures in regenerative medicine. Harvesting MPCs from a tonsillar biopsy reduces the need for a major surgical site and is a procedure that can be performed in an outpatient setting, thus having less morbidity compared to bone marrow biopsy or extensive liposuction. Furthermore, it does not rely on discarded tissues from unrelated surgical procedures.

## Conclusions

Isolation and culture expansion of adult stem/progenitor cells is a critical step in cell therapy. A large number of stem cells are required for therapeutic uses. Recent studies have shown that MPCs from different anatomical sites differ widely in expression signatures and differentiation potency [24]. Therefore, the tissue source and the derivation process determine the abundance, phenotype, and differentiation potency of MPCs [3, 11–13, 25]. Our novel procedure achieves a high yield of tonsillar biopsy-derived MPCs. We demonstrate their high expansion potential through at least 40 population doublings within a short time. Therefore, our studies indicate that tonsillar biopsies smaller than 1 g of tissue are an excellent, translational source of MPCs for research and clinical applications.

## Additional file

Additional file 1: Supplementary Table 1. (XLSX 12 kb)

## Abbreviations

BM: Bone marrow
BSA: Bovine serum albumin
CM: Complete medium
DMEM: Dulbecco’s modified Eagle’s medium
DPBS: Dulbecco’s phosphate-buffered saline
FBS: Fetal bovine serum
GFP: Green fluorescent protein
IgG: Immunoglobulin G
IMDM: Iscove’s modified Dulbecco’s medium
IRB: Institutional Review Board
MPC: Mesenchymal progenitor cell
ODM: Osteoblast differentiation medium
P: Passage
PBS: Phosphate-buffered saline
PCR: Polymerase chain reaction
PD: Population doublings
T-MPC: Tonsillar mesenchymal progenitor cell
